# Weaning from Kidney Replacement Therapy in the Critically Ill Patient with Acute Kidney Injury

**DOI:** 10.3390/jcm13020579

**Published:** 2024-01-19

**Authors:** Kada Klouche, Vincent Brunot, Romaric Larcher, Alexandre Lautrette

**Affiliations:** 1 Intensive Care Unit Département, Lapeyronie University Hospital Montpellier, 34295 Montpellier, France; v-brunot@chu-montpellier.fr (V.B.); r-larcher@outlook.fr (R.L.); 2 Phymedexp, Faculty of Medicine, Université de Montpellier, Inserm, Centre National de Recherche Scientifique (CNRS), CHRU de Montpellier, 34295 Montpellier, France; 3 Centre de Lutte Contre le Cancer Jean PERRIN, Médecine Intensive Réanimation, CHU Clermont-Ferrand, 63000 Clermont-Ferrand, France; alexandre.lautrette@clermont.unicancer.fr

**Keywords:** acute kidney injury, kidney replacement therapy (KRT), KRT weaning, urine output, creatinine clearance, urinary urea, urinary creatinine, urinary biomarkers, critical care

## Abstract

Around 10% of critically ill patients suffer acute kidney injury (AKI) requiring kidney replacement therapy (KRT), with a mortality rate approaching 50%. Although most survivors achieve sufficient renal recovery to be weaned from KRT, there are no recognized guidelines on the optimal period for weaning from KRT. A systematic review was conducted using a peer-reviewed strategy, combining themes of KRT (intermittent hemodialysis, CKRT: continuous veno-venous hemo/dialysis/filtration/diafiltration, sustained low-efficiency dialysis/filtration), factors predictive of successful weaning (defined as a prolonged period without new KRT) and patient outcomes. Our research resulted in studies, all observational, describing clinical and biological parameters predictive of successful weaning from KRT. Urine output prior to KRT cessation is the most studied variable and the most widely used in practice. Other predictive factors, such as urinary urea and creatinine and new urinary and serum renal biomarkers, including cystatin C and neutrophil gelatinase-associated lipocalin (NGAL), were also analyzed in the light of recent studies. This review presents the rationale for early weaning from KRT, the parameters that can guide it, and its practical modalities. Once the patient’s clinical condition has stabilized and volume status optimized, a diuresis greater than 500 mL/day should prompt the intensivist to consider weaning. Urinary parameters could be useful in predicting weaning success but have yet to be validated.

## 1. Introduction

Acute kidney injury (AKI) is a common pathology in critical care settings, affecting more than half of all patients, 10% of whom require kidney replacement therapy (KRT) [1,2,3]. Modalities of KRT currently available include intermittent hemodialysis and continuous renal replacement therapies (continuous veno-venous hemodialysis or hemo/dia/filtration). Though a better survival using continuous compared with intermittent RRT has not been evidenced [4,5,6,7], the former has gained wide application in ICUs, often supplanting intermittent modalities because of the belief that it is better tolerated in hemodynamically unstable patients [8]. Regardless of the modality used, the need for KRT considerably increases in-hospital mortality, which then fluctuates between 40% and 60% [9,10]. More than three-fourths of patients who survive this acute episode develop chronic renal failure, 10 to 30% of whom remain dependent on KRT [11,12,13,14,15]. In the long term, they remain exposed to a worsening of their morbidity and mortality, and a deterioration in their quality of life [15,16,17].

In severe forms of AKI, the indication for initiating KRT has been the subject of several studies, leading to a consensus that is now widely shared [18,19,20,21]. On the other hand, weaning from KRT has been little studied. Indeed, a majority of surviving patients recover satisfactory renal function, but there is no consensus or standardized attitude guiding the discontinuation of KRT [11,22]. The KDIGO guidelines suggest weaning from acute KRT when it is no longer required after sufficient renal recovery has occurred and meets patient’s needs or because KRT is no longer consistent with the goals of care [23]. The lack of specificity of these statements indicates that clinical or functional markers that may help physicians to predict successful cessation of acute KRT are still lacking. KRT exposes the patient to several complications, and its prolonged continuation could also be deleterious for renal recovery. However, premature cessation of KRT may re-expose the patient to all of the complications of AKI, including fluid overload with delayed weaning from the ventilator, electrolyte and acid–base disorders and uremia. Consequently, early and successful weaning from KRT should be one of the main objectives of critical care management of AKI.

In this review, we will outline the evidence in favor of early weaning, the tools available to predict success, and the practicalities of weaning from KRT. With this in mind, we reviewed the main studies, most of them observational, that have investigated KRT weaning in the critically ill patient to identify the main findings. Although the modality of KRT weaning is far from being validated, this review proposes a practical attitude that needs to be confirmed by future randomized studies.

## 2. Reasons for Optimal and Earliest KRT Weaning

In the management of AKI, the indication for KRT depends on the severity of renal damage and metabolic disorders. Once initiated, KRT is prolonged for an average of 5 to 10 days, after which the intensivist must temporarily interrupt treatment in order to assess native kidney function and attempt weaning [24]. These interruptions may, however, come too late or too early.

**Figure 1:**
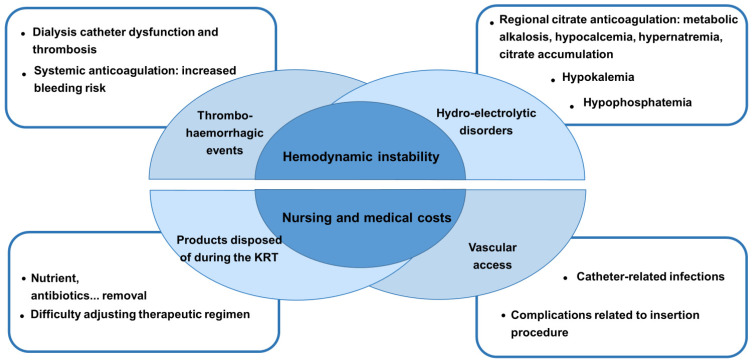
Complications and burden associated with Kidney Replacement Therapy (KRT).

KRT exposes patients to numerous complications, such as vascular access-associated infections or thrombosis, bleeding favored by systemic anticoagulation, drug and antibiotic elimination, electrolyte (phosphorus) and nutrient depletion, hemodynamic instability and pro-inflammatory effects generated by extracorporeal circulation [9,10]. The reported risk for bacteremia with nontunneled percutaneous catheters ranges between 3% and 10% in the ICU, increasing significantly after 1 week of catheter use [25,26,27]. This daily risk of colonization and catheter-related infections was reported to be significantly higher in dialysis catheters compared to central venous catheters within the first 7 days of catheter maintenance [28]. The incidence of thrombosis of a cannulated vein ranged from 20% to 70%, depending on the site and diagnostic procedure [29]. In a prospective study, vascular access thrombosis was observed in 2.3 to 4.2 episodes per 1000 days of temporary catheters [30]. During KRT in critically ill patients, adverse events occurred in 23% and 16% of patients, respectively, in each group analyzed in the STARRT-AKI study (evaluating an accelerated vs. standard KRT strategy). Hypotension and hypophosphatemia were the most frequent adverse events [20]. The occurrence of intradialytic arterial hypotension, sometimes subclinical, is thought to lead to renal ischemic lesions [31]. Indeed, renal biopsies carried out in patients who had been on hemodialysis for several days or weeks revealed recent tubular ischemic lesions probably induced by per-dialytic hypotensive episodes [32]. Similar observations were reported by Conger [33], who also demonstrated, in an experimental model of post-ischemic AKI, a loss of renal plasma flow autoregulation, explaining the vulnerability of renal tubular cells to even small drops in blood pressure. He showed, in a rat model of ischemic AKI, that a reduction in renal perfusion pressure within the autoregulatory range induced a marked decrement in renal blood flow in AKI rats compared to that of controls. These renal ischemic micro-lesions would delay the recovery of renal function [34]. The elimination of mediators and growth factors required for tubular cell regeneration may also contribute [35]. An untimely increase in the dose of dialysis delivered could also affect renal recovery. A meta-analysis found that intensification of KRT was associated with an increase in dependence on this therapy [36]. The prolongation of the duration of AKI is also associated with a worsening of mortality [24]. These observations suggest that unwarranted prolongation of KRT is deleterious. It would also unnecessarily increase workload and treatment cost (Figure 1). On the other hand, stopping KRT too early exposes the patient to fluid overload, with its consequences for ventilation, electrolyte and acid–base disorders, and nitrogen retention. Indeed, elevated blood urea could lead to adverse effects such as digestive hemorrhage. Aggravation of metabolic acidosis due to failure of the native kidney to eliminate acids, and discontinuation of KRT, is deleterious. Finally, the risk of fluid overload remains, with its cardiopulmonary impact. If weaning fails, KRT must be repeated, with new vascular access and all of the complications already described. Wu et al. [37] reported that re-institution of KRT after weaning failure significantly worsened the prognosis, without, however, being able to distinguish between the severity of the disease and the impact of the weaning attempt. They retrospectively studied 304 postoperative patients who had undergone KRT. A third of their patients (94, 30.9%) were weaned off acute dialysis for more than 5 days, and 64 (21.1%) were successfully weaned for at least 30 days. They found that surgical patients with AKI requiring resumption of dialysis after being temporarily weaned had a worse prognosis. Other observational studies also suggest that weaning failure is associated with increased mortality [38,39,40]. Whether failure of weaning from KRT is harmful by itself or just a marker of severity of disease remains, however, questionable.

Thus, as soon as an KRT is initiated, it should be possible to envisage its cessation as early as possible and under the best possible conditions [41].

## 3. Predictive Criteria for Successful Weaning from KRT

The international KDIGO recommendations, dating from 2012, suggest weaning the patient off KRT when it is no longer necessary due to sufficient renal recovery to meet the patient’s needs, or because it is no longer consistent with the goals of care [23]. The generality of this statement underlines the lack of objective data that would make it possible to protocolize weaning [41]. Nevertheless, several strategies are practiced, ranging from a “late” to an “early” attitude, with the risk of unjustified prolongation or unsuccessful attempts in either case.

**Figure 2:**
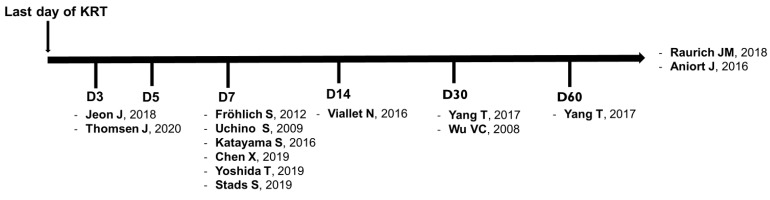
Different definitions of KRT weaning (number of days without KRT) used in the literatureFor each weaning period, the relative study and reference are shown in brackets KRT: Kidney replacement therapy [37,38,39,42,43,44,45,46,47,48,49,50,51].

Most studies of weaning from KRT are observational and vary in terms of quality and definition of success. Successful weaning is defined by the cessation of KRT, the duration of which may vary between 7 and 28 days, depending on the study, during which time serum creatinine levels should fall or only stabilize (Figure 2).

The first criterion that could justify discontinuation of KRT is the resumption of diuresis. A survey of intensivists in England showed that an increase in diuresis was the most frequently cited reason for weaning (74%), followed by normalization of pH (70%) and achievement of adequate hydration (55%) [52]. In a case–control study including 304 patients treated with intermittent KRT, 94 (31%) were weaned for at least 5 days and 64 (21.1%) for at least 30 days. Longer duration of KRT, higher SOFA score and diuresis less than 300 mL/24 h on the day of the weaning attempt, and age > 65 years were predictive of failure before day 30 [37]. An international study included 1006 patients on continuous KRT, of whom 529 survived and 313 were successfully weaned from KRT for more than 7 days. The mortality of those weaned was significantly lower than that of others (28.5% vs. 42.7%, *p* < 0.0001) [38]. The best predictor of weaning success was diuresis, with sensitivity and specificity optimal for a threshold of 436 mL/24 h without diuretics and 2330 mL/24 h with diuretics. Other studies have confirmed the strong predictive value of diuresis in weaning success [39,40,42,43,44,45]. The thresholds observed in the aforementioned study [38], by collecting diuresis 24 h before discontinuation of the KRT, appear to be those adopted by the majority of authors [53].

However, weaning based on urine volume alone is no guarantee of success. The use of a weaning algorithm based on the presence of a diuresis of more than 500 mL/24 h enabled only one-third of eligible patients to be weaned, and KRT was continued in 69% of patients because of fluid overload [54]. The significant increase in urine output obtained after diuretics simplifies the management of the hydro-sodic status, but it is difficult to interpret and to associate with renal recovery, as the scant data are sometimes contradictory [53]. Despite controversial reports, the use of diuretics is often associated with successful weaning [38,43]. In a prospective analysis of 92 patients, fluid–sodium balance in the 48 h following discontinuation of KRT was negative in successfully weaned patients at D7, whereas it was largely positive in those who had failed, with no significant difference in diuretic use between the two groups [46]. However, a comparison of furosemide (0.5 mg/kg/h) with placebo after continuous KRT failed to show any benefit in terms of time to renal recovery [55].

Assessment of glomerular filtration rate (creatinine clearance) is the best marker of possible recovery of function. It needs to be carried out in a steady-state situation, which is difficult to obtain, particularly in the case of intermittent techniques inducing rapid and significant variations in solute concentrations. Given the reliability of the assessment only during the inter-dialytic period, clearance measurements (UV/P) have been proposed for relatively short periods ranging from 2 to 12 h. In a retrospective analysis of 53/85 weaned patients, creatinine clearance by two-hour urine collection outperformed diuresis in predicting weaning success [47]. A clearance greater than 23 mL/min, in the 12 h preceding cessation of continuous KRT, had the best sensitivity, specificity and positive predictive value for weaning up to D7. In the ATN study [56], it was recommended to stop RRT as soon as diuresis exceeded 30 mL/h and if creatinine clearance over 6 h was >20 mL/min, to continue it if it was <12 mL/min, and to leave the choice to the intensivist between these two values. In the course of inclusion, practice has shown that the threshold of 20 mL/min is too high, leading to a new amendment recommending a lowering of this threshold to 12 mL/min [57]. These data can be compared with those of a prospective study that showed that creatinine clearance > 11 mL/min or a reduction in creatinine levels between D_0_ and D_2_ were associated with successful weaning at D_7_ [46]. However, measuring clearance requires accurate urine collection, which may be difficult to achieve and overestimates glomerular filtration rate due to tubular secretion of creatinine [58].

Biochemical analysis of simple urinary markers such as urea and creatinine could be of interest in assessing renal excretory function. Retrospective analysis of 54 patients who survived severe AKI showed that creatininuria ≥ 5.2 mmol/24 h on D_1_ of KRT discontinuation, irrespective of diuretic use, was associated with successful weaning in 84% of cases (not requiring RRT within 15 days of KRT discontinuation) [48]. In a similar study involving patients treated with intermittent KRT, a urine urea > 1.35 mmol/kg/24 h predicted weaning success with an AUC of 0.96, significantly better than a urine output > 8.5 mL/kg/h [49].

The new blood and urine biomarkers that have been developed for the early diagnosis of AKI [59,60] have, for some at least, been tested in the assessment of renal recovery. Several urinary biomarkers, including NGAL, HGF, KIM1, Cys-C and [TIMP-2]x[IGFBP7], that correlate with renal cell injury or function could predict renal recovery and outcome of AKI. Indeed, studies in critically ill AKI patients treated with KRT have shown that those with lower initial levels of biomarkers of inflammation and tissue or kidney injury or whose levels of these biomarkers decrease over time are more likely to recover kidney function [61,62]. Also, plasma NT-pro-BNP at the initiation of CKRT has been found to be a weaning-related factor [63]. However, these studies focused more on the differentiation between transient and persistent AKI and on renal recovery than on predictive value for successful KRT cessation. Only a prospective study including 110 AKI patients treated by CKRT showed that serum CysC (less than 1.85 mg/L) was an independent predictor of the successful weaning from CKRT for more than 14 days [40]. In contrast, in a prospective study of 54 patients, urinary NGAL did not outperform urine output in predicting successful weaning from KRT at 72 h. This performance was, however, improved by combining 24-h diuresis and NGAL levels at H6 of weaning [50]. Stads et al. [46] confirmed these observations and found the performance of urinary NGAL to be inferior to that of creatinine clearance at D_2_. Kim et al. [40] found no significant association between plasma NGAL levels and successful weaning from KRT. In a recent study, a plasma NGAL level ≤ 403 ng/mL was predictive of successful weaning from continuous KRT in non-septic patients, with diuresis being more informative in septic patients [39]. Plasma Cystatin C levels at initiation were a factor associated with successful weaning [64] and were associated with a better long-term renal prognosis when <2.97 mg/L at KRT discontinuation [51].

Due to the heterogeneity of studies and proposed thresholds, it is difficult to conclude on the real usefulness of plasma Cystatin C [53]. In short, current data do not support the use of biomarkers to guide the discontinuation of RRT. In fact, they were evaluated in the assessment of renal recovery and not in the weaning from KRT.

Table 1 summarizes the advantages and disadvantages of the different KRT weaning criteria.

## 4. Practical Procedures for Weaning off Renal Replacement Therapy

Currently, practices of KRT weaning depend on every center policy and vary from a “wait and see” to a “go fast” attitude. The “wait and see” attitude may prolong KRT needlessly and expose the patient to its hazardous effects without any benefit. Conversely, a “go fast” attitude may lead to several unsuccessful attempts of weaning, with the subsequent requirement of reinstitution that is by itself harmful. Appropriate cessation of KRT is obviously critical to clinical and economic outcomes, but data on how and when to stop KRT in the critically ill and identification of predictive factors of successful cessation of KRT remain under scrutiny. Indeed, no prospective randomized trials exist for the cessation of KRT [57], whereas weaning from mechanical ventilation, a crucial aspect of pulmonary support, has been extensively investigated [65].

Before considering weaning from KRT, the following conditions must be met: (1) the cause of the AKI and precipitating factors are identified and resolved. (2) Hemodynamic and respiratory stability of the patient is achieved. (3) The patient’s fluid status is optimized. (4) Initiation of diuresis is observed. It is important to consider the patient’s clinical condition, comorbidities and volemic status before initiating KRT weaning. In a retrospective study of 316 patients with AKI, the absence of comorbidities such as chronic kidney disease (CKD), cardiovascular disease and hypertension at the time of KRT indication and successful discontinuation were identified as variables associated with hospital discharge [66]. Among these, the most important preexisting risk factor for non-recovery from AKI is CKD. The prevalence of CKD among critically ill patients with AKI is approximately 30% and is likely to increase in the context of an aging population [67]. Lower premorbid function is more predictive of nonrecovery and dialysis dependence probably because it indicates poor renal reserve to the acute stress of critical illness and AKI [67]. In addition, the patient’s hydration status during the KRT weaning trial must be optimal. The volume elimination requirement must not exceed the daily urine output, otherwise the occurrence of pulmonary edema could compromise KRT cessation [68].

Once a return to diuresis has begun, weaning from KRT must be addressed. In the case of intermittent KRT, it is easy to space out the sessions so as to be able to assess diuresis, analyze urine, assess the natural evolution of creatinine levels to identify the onset of a plateau, and if necessary, measure creatinine clearance. In the case of a continuous modality, you need to be able to spot an unexplained drop in creatinine levels and program an interruption of treatment.

The diuresis threshold above which weaning can be attempted has not been established with certainty. All authors agree that this threshold should be set at a urine volume > 500 mL/24 h (or 20–25 mL/h) without diuretics, and >2000 mL/24 h with diuretics. At this stage, KRT sessions are stopped to monitor a continuous and progressive improvement in renal function. KRT will only be resumed if there are urgent indications similar to those for initiating KRT (Table 2).

A more proactive approach is to measure native clearance over 2 to 24 h at the diuresis levels described above. Clearance > 12–15 mL/min is predictive of successful weaning. Assessing urinary excretion and concentration capacity by measuring urinary creatinine and urea would be a simpler and more cost-effective alternative. An approach based on diuresis and creatininuria has been proposed [69]. It includes clearance and urinary thresholds for urea and creatinine (1.35 mmol/kg/24 h and 5.2 mmol/24 h, respectively). Although not fully validated, this strategy has the merit of prompting the intensivist to ask the question of weaning at an early stage, and to take an interest in the biochemical analysis of urine during the recovery phase.

**Figure 3:**
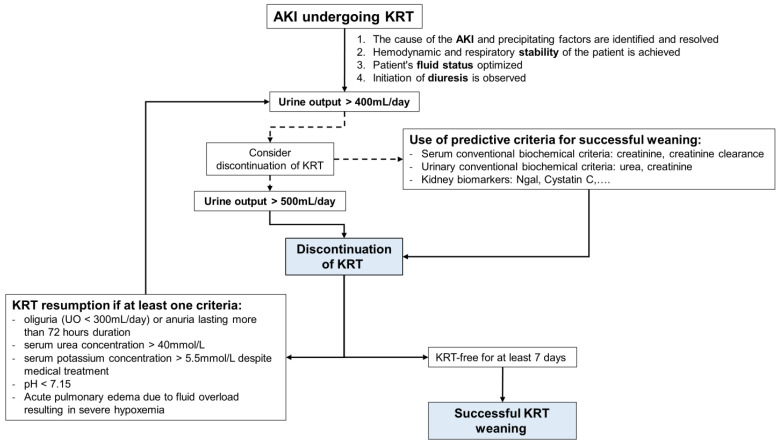
A decision algorithm for weaning from Kidney Replacement Therapy (KRT).

A decision algorithm for weaning from KRT is shown in Figure 3 for illustrative purposes.

## 5. Future Developments

Recovery of renal function is and will remain one of the major challenges in the management of AKI. Predicting the appropriate time for weaning from extracorporeal depuration should integrate various clinical and biological parameters, including premorbid conditions. Urine output and creatinine clearance are commonly used for this purpose, but their value needs to be studied further, along with the contribution of new urinary and plasma biomarkers. Indeed, few studies have been able to identify the ideal markers of sufficient recovery of renal function to avoid resetting the KRT. Future work should incorporate evolving data on new biomarkers of kidney damage and repair and bedside measurements of real-time GFR. Large multicenter randomized trials are definitely needed to definitively guide appropriate discontinuation.

The ultimate goal should be recovery of renal function, but also restoration of functional status to pre-morbidity levels. In addition, the assessment of renal function after weaning must take into account the loss of muscle mass and its impact on serum creatinine. The use of other GFR markers that are not sensitive to muscle mass (e.g., cystatin C) or direct quantification of GFR should be considered.

## 6. Conclusions

Kidney replacement therapy for AKI marks a turning point in the prognosis and evolution of this pathology. As soon as KRT is initiated, weaning should be one of the intensivist’s main preoccupations, and care should be taken not to delay it or bring it forward prematurely. Once the patient’s clinical condition has stabilized and volume status optimized, a diuresis greater than 500 mL/day should prompt the intensivist to consider weaning. The resumption of, or increase in, diuresis is a necessary, but not sufficient, condition for discontinuing KRT sessions. Analysis of urinary excretion of urea and creatinine, combined with quantification of diuresis, probably significantly improves prediction of weaning success. Other variables, notably urinary and serum renal biomarkers (Cystatin C, N gal, KIM 1, etc.) have shown potential in predicting CRRT weaning success, but the available studies are limited by their design, the heterogeneity of the variables and the lack of prospective validation. Nevertheless, weaning from KRT, like weaning from artificial ventilation, should be protocolized on the basis of current knowledge and the results of future randomized multicenter trials. Henceforth, the management of AKI in the critical patient should obey the following adage: “less and better kidney replacement therapy in acute kidney injury”.

## Figures and Tables

**Table 1:** Advantages and disadvantages of predictors for successful kidney replacement therapy discontinuation.

Urine Output		Serum Conventional Biochemical Criteria (Serum Creatinine, Creatinine Clearance, Kinetic eGFR, eGFR)		Urinary Conventional Biochemical Criteria (24 h Urine Creatinine, Daily Urinary Urea Excretion)		Kidney Biomarkers (Serum Cystatin C, NGAL, IL-18, IL-6, Proenkephalin)	
Advantage	Disadvantage	Advantage	Disadvantage	Advantage	Disadvantage	Advantage	Disadvantage
Simple and reliable collection	Dependent on hydration state	Simple, accurate and reproducible measurement	Requires stabilization of creatinine value	Robustness of rationale	Possible impact of diuretics, protein input and hypercatabolism	High sensibility	Several cut-off values
Low cost	Dependent on hemodynamic state			Simple, accurate and reproducible measurement		Used in diagnosis, severity, and prognosis of AKI	Requires stabilization of value for biomarkers partially removed by RRT
	Several cut-off values with or without diuretics						Impact of other diseases on value (ex: sepsis)
							More expensive
							Not available in all centers

**Table 2:** Criteria for resuming kidney replacement therapy (KRT) after weaning cessation.

KRT Resumption If One of the Following Criteria Occurs:
Oliguria (UO < 300 mL/24 h) or anuria lasting more than 72 h in duration
Serum urea concentration > 40 mmol/L
Serum potassium concentration > 5.5 mmol/L despite medical treatment
pH < 7.15 in a context of pure metabolic or mixed acidosis despite medical treatment
Acute pulmonary edema due to fluid overload resulting in severe hypoxemia requiring oxygen flow rate > 5 L/min to maintain an SpO_2_ ≥ 95% or requiring FiO_2_ > 50% despite diuretic therapy

## Data Availability

Not applicable.
